# Gamma low field magnetic stimulation ameliorates pathophysiological damage and cognitive impairments in AD mice

**DOI:** 10.1186/s13195-026-02052-1

**Published:** 2026-04-18

**Authors:** Weiran Zheng, Duyan Geng, Alan Wang, Zhichao Li, Aoge Liu, Shuting Chen, Qiang Wang, Chi Su, Ziyi Yan, Yue Yin, Guizhi Xu

**Affiliations:** 1 School of Electrical Engineering, Hebei University of Technology, Tianjin, 300401 China; 2 State Key Laboratory of Smart Power Distribution Equipment and System, Hebei University of Technology, Tianjin, 300401 China; 3 Innovation and Research Institute of Hebei University of Technology in Shijiazhuang, Shijiazhuang, 050299 China; 4 Auckland Bioengineering Institute, University of Auckland, Auckland, 1010 New Zealand; 5 Shijiazhuang Dukon Medical Instruments Co., Ltd, Shijiazhuang, 050081 China; 6 School of Health Science and Biomedical Engineering, Hebei University of Technology, Tianjin, 300130 China

**Keywords:** Alzheimer's disease, Gamma low field magnetic stimulation, Hippocampal CA1 region, Neural network properties, Amyloid-beta 42, Cognitive

## Abstract

**Background:**

The normal functioning of gamma rhythms is crucial for maintaining brain health, while their abnormalities are closely associated with various neurological disorders, particularly Alzheimer’s disease. Gamma stimulation modalities including auditory, visual, electrical, and strong magnetic approaches have all demonstrated potential therapeutic effects for AD, with substantial research findings continuously emerging. However, 40 Hz gamma low field magnetic stimulation(gamma-LFMS) remains unexplored.

**Methods:**

To investigate this question, we applied pulsed magnetic fields with a magnetic field strength of 10 mT and frequency of 40 Hz (2 × 30 min/day) to 9-month-old APP/PS1 double transgenic AD model mice for 18 consecutive days, and evaluated changes in spatial memory capacity, hippocampal neural network characteristics, and amyloid protein 42 content in AD mice.

**Results:**

Gamma-LFMS significantly enhanced spatial memory performance in AD mice, increased theta-gamma phase-amplitude coupling and gamma band power in the hippocampal CA1 region, showed a trend toward desynchronization in low gamma, and effectively reduced hippocampal β-amyloid42 burden.

**Conclusions:**

This study demonstrates for the first time that gamma-LFMS effectively ameliorates pathophysiological alterations and spatial memory deficits in AD mice. These findings address a critical knowledge gap regarding the effects of gamma-LFMS on AD pathology and provide a theoretical foundation for developing cost-effective home-based prevention and treatment devices applicable throughout the lifespan.

**Supplementary Information:**

The online version contains supplementary material available at 10.1186/s13195-026-02052-1.

## Introduction

Alzheimer’s disease (AD) constitutes the primary type of dementia, comprising 60%–70% of dementia diagnoses globally [1]. Worldwide statistics suggest that over 55 million individuals are currently living with AD and related dementia conditions [2], which has become a major issue affecting global public health and sustainable social development. The principal features of AD include gradual memory deterioration and cognitive dysfunction, with key pathological processes centered on irregular accumulation of β-amyloid protein (Aβ) and excessive phosphorylation of tau protein [3]. Presently, therapeutic approaches for AD patients primarily concentrate on symptom alleviation and employing targeted medications to manage brain tissue damage, yet these strategies fail to prevent disease advancement, with effective treatment interventions remaining limited [4]. Gamma rhythm (30–80 Hz) stimulation improves AD pathology by restoring impaired neural oscillations, providing new insights and discoveries for pathological treatment of AD [5, 6]. However, current research predominantly employs optical [7–10], acoustic [5, 11, 12], electrical [13–16], and high-intensity gamma rhythm magnetic stimulation [17], while whether gamma-LFMS can intervene in AD remains unexplored.

Among studies investigating gamma rhythm stimulation, 40 Hz has demonstrated prominent effects. For instance, the Martorell et al. [18] found that 40 Hz non-invasive light flickering stimulation and auditory stimulation could enhance endogenous gamma rhythm neural rhythmic activity within the visual and auditory cortical regions of AD mice through entrainment mechanisms, respectively, and significantly reduce Aβ in both brain regions. Additionally, both stimulations enhanced gamma rhythm neural oscillations in hippocampal CA1. When both stimulations were applied synchronously to AD mice, they activated the brain glymphatic system by enhancing gamma rhythm neural oscillations and promoted Aβ clearance through increased flow of cerebrospinal fluid and interstitial fluid [5]. 40 Hz transcranial alternating current stimulation (tACS) and transcranial magnetic stimulation (TMS) likewise enhance gamma rhythm neural oscillations in the brain [16, 17, 19], but due to electromagnetic interference, synchronous recording of brain neural rhythm signals during stimulation is not feasible, and direct entrainment phenomena were not observed in post-stimulation recordings. Current research on 40 Hz TMS enhancing brain gamma rhythm neural oscillations is limited and generates very high magnetic field intensities (approximately 1 Tesla) [17]. Notably, gamma rhythm stimulation via light and sound modalities compromises patient compliance during treatment and may induce visual and auditory damage. Electrical stimulation is contingent upon electrode placement, while high-intensity magnetic stimulation involves complex operations and poses challenges for device miniaturization. These factors present obstacles to gamma rhythm stimulation therapy for AD. In contrast to acoustic, optical, electrical, and high-field magnetic stimulation approaches, low-field magnetic stimulation can circumvent these limitations. However, whether gamma-LFMS can ameliorate gamma rhythm impairments and cognitive deficits in AD mice remains unclear.

In this study, we employed Morris water maze (MWM) testing, resting-state hippocampal CA1 local field potential (LFP) analysis, and Aβ immunofluorescence detection to investigate the effects of gamma-LFMS on pathophysiology and spatial memory in AD mice. Our findings initially indicate that gamma-LFMS can significantly enhance gamma rhythm power and modulate neural network synchrony within the hippocampal brain region of AD mice, effectively clear Aβ, and improve spatial memory capacity.

## Materials and methods

All experimental operations in this work were carried out in compliance with the Guide for the Care and Use of Laboratory Animals [20]. All experimental schemes were reviewed and approved by the Biomedical Ethics Committee of Hebei University of Technology (approval number: HEBUTaCUC2022043).

### Experimental animals

This study utilized a total of 24 mice (9 months old, 12 males and 12 females), comprising 8 wild-type C57BL/6J mice and 16 homologous APP/PS1 double transgenic mice (ADS: *n* = 8; gamma-LFMS: *n* = 8). All experimental animals weighed between 20 and 25 g and were purchased from Beijing Huafukang Bioscience Co., Ltd. Animals were reared under standard laboratory conditions (day/night cycle of 12 h, ambient temperature 22 ± 2 °C, air humidity 50 ± 5%) with food and water ad libitum.

### Gamma-LFMS system and parameters

**Fig. 1:**
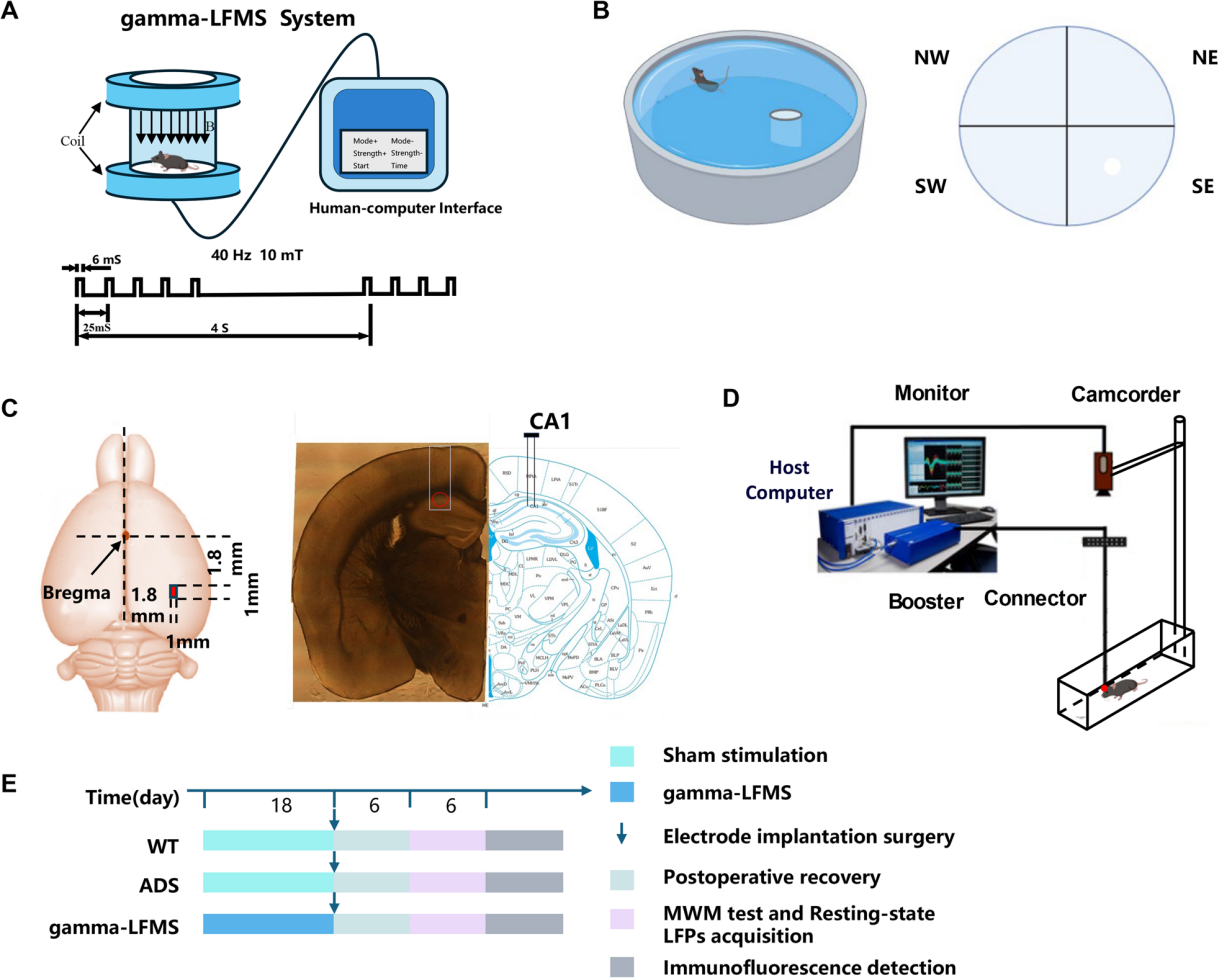
Schematic diagram of experimental design. **A** Schematic representation of the gamma-LFMS system and magnetic field pulse waveform; (**B**) Schematic diagram of the Morris water maze test in mice; (**C**) Schematic illustration of multi-channel microelectrode implantation; (**D**) Local field potential (LFP) signal acquisition system; (**E**) Experimental workflow diagram

This study employed a custom Helmholtz coil system to generate precisely controlled gamma-LFMS. The system consisted of a digital control unit and a pair of precision Helmholtz coils that produced a highly uniform magnetic field within the target region. The coil geometric parameters were: interior diameter 200 mm, outer diameter 260 mm, coil separation 130 mm (0.65 × inner diameter, conforming to Helmholtz configuration standards), ensuring magnetic field uniformity within ± 3% in the central exposure area. The system was equipped with temperature-controlled cooling fans to maintain coil temperature at 23 ± 1 °C during exposure, eliminating thermal interference. Magnetic field parameters were set as follows: peak amplitude 10 mT, frequency 40 Hz, rectangular waveform, duty cycle 24%, burst mode (burst duration comprising 33% of total cycle). Field strength was calibrated using a gaussmeter (Model 5180, F.W. Bell Inc., USA) prior to experiments to ensure precise and controllable exposure dosage. The gamma-LFMS group AD mice received two sessions of stimulation daily, each lasting 30 min, for 18 consecutive days. The sham stimulation group (ADS) underwent identical handling procedures as the stimulation group, but without powering the stimulation device. All experiments were conducted under identical experimental conditions (ambient temperature 22 ± 2 °C, air humidity 50 ± 5%) to eliminate potential confounding factors. Thermal imaging technology was employed throughout to monitor animal cranial temperature, confirming no significant heating effects (temperature change < 0.2 °C). Figure 1A illustrates the gamma-LFMS system and pulse waveform characteristics. To assess whether gamma-LFMS could reach deep structures, we computed the spatial distribution of the stimulation-related field in a mouse brain model using COMSOL based on the coil geometry and experimental positioning. The hippocampal CA1 location was determined using segmentation at the corresponding anatomical level. The simulated field map indicates that the induced electric field magnitude (|E|, V/m) to the depth of the hippocampus and covers the CA1 region under the present stimulation parameters (Fig. S1).

### Spatial memory assessment

Spatial learning and memory capabilities of mice were evaluated using the Morris water maze (MWM) [21]. This established paradigm efficiently differentiates cognitive function from motor performance and has been extensively utilized for spatial navigation and memory evaluation in rodents. The experimental setup comprised a stainless steel circular tank (diameter 120 cm, height 50 cm) containing water at 30 cm depth, with water temperature controlled at 23 ± 2 °C to eliminate thermal stress influences on behavioral outcomes. The tank was conceptually divided into four equivalent quadrants (northwest, northeast, southwest, southeast), with a transparent acrylic platform (diameter 10 cm, height 28 cm) placed at the southeast quadrant center, 2 cm beneath the water surface. Remote visual markers were positioned at consistent locations surrounding the maze to supply stable spatial landmarks. All experiments were documented and evaluated using a video tracking system (ANY-maze, Stoelting Co.). The training regimen encompassed six successive days of spatial learning trials, with four trials per mouse per day. For each daily session, mice were gently placed into the water from the center of each of the four quadrants (one trial per quadrant) in a randomized order. Each session lasted a maximum of 60 s; subjects were required to stay on the platform for 15 s following discovery to reinforce spatial memory. When subjects failed to identify the platform within the designated timeframe, they were gently directed to the platform and provided the identical 15 s platform exposure. Inter-trial intervals were 30 s to minimize proactive interference effects. After each test, animals were gently dried with absorbent towels and returned to their housing cages (Fig. 1B). Primary outcome measures included: (1) escape latency (seconds): the average value of the four daily trials, representing the time required for animals to locate the platform and reflecting spatial learning ability; (2) swimming speed (cm/s): serving as a control variable to exclude potential confounding factors arising from motor function differences.

### Local field potentials signal recording and analysis

Following completion of gamma-LFMS and behavioral testing, electrode implantation surgery was performed in the hippocampal CA1 region to record neural oscillatory activity (Fig. 1C). The surgical procedure was adapted from reference [22] and is briefly described as follows: mice were anesthetized with isoflurane and secured in a stereotaxic apparatus (David Kopf Instruments, USA). After exposing the skull, 3–4 stainless steel anchor screws were implanted in non-target regions to provide structural support and electrical grounding. Craniotomy was performed over the hippocampal CA1 region (anteroposterior: -1.8 to -2.8 mm, mediolateral: -1.8 to -2.8 mm relative to bregma). Nichrome electrodes (35 μm diameter) were implanted into the target brain regions (dorsoventral: 2 mm) using microdrives (Plexon Inc., USA) and secured with dental cement. LFPs recordings were conducted 7 days post-surgery to allow for recovery. Resting-state brain electrical activity was recorded for 30 min in a quiet (Fig. 1D), familiar environment using an OmniPlex/128 multichannel acquisition system (Plexon Inc.), and LFPs for subsequent analysis were acquired at a sampling rate of 1000 Hz, following amplification (gain: 5000) and low-pass filtering (0.3–300.0 Hz). Signal preprocessing included baseline drift removal using fifth-order Coiflet wavelet decomposition and a 50 Hz notch filter to eliminate electrical interference. Continuous recordings were segmented into 4-second epochs for subsequent spectral analysis. To characterize the dynamic properties of hippocampal oscillations, the following metrics were analyzed: (1) Power spectral density (PSD): calculated using Welch’s method (Hamming window, 50% overlap); (2) Phase–amplitude coupling (PAC) was quantified using the method proposed by Tort et al., implementing a mutual information–based modulation index (MI) [23, 24] to characterize interactions between theta (4–12 Hz) phase and gamma (30–100 Hz) amplitude. Specifically: (i) theta phases φ(t) were obtained by applying a narrowband FIR band-pass filter with a 1 Hz bandwidth and 1 Hz step size, and the resulting phase time series were divided into 16 equally spaced bins (22.5° per bin); (ii) high-gamma activity was extracted using a sliding 20 Hz-wide band-pass filter (2 Hz step size), and the corresponding amplitude envelopes A(t) were derived via the Hilbert transform; (iii) the MI was computed by normalizing the mean high-gamma amplitude within each phase bin to form a probability distribution, and 10 surrogate datasets were generated to identify and exclude spurious coupling; (3) Phase-locking value (PLV) was used to quantify inter-channel phase synchronization [25]. Signals were filtered into theta and gamma bands using the same filtering parameters as in the PAC analysis, and instantaneous phases were obtained via the Hilbert transform. PLV was then calculated as the magnitude of the circular mean of the complex exponentials of the phase differences, yielding values between 0 (no phase locking) and 1 (perfect phase locking).

### Immunohistochemical analysis

Immunofluorescence techniques were employed to detect Aβ42 deposition in brain tissue. The processing workflow for paraffin-embedded brain sections was as follows: sections were first deparaffinized in Dewaxing/Clearing Reagent for Histology (Servicebio Biotechnology Co., Ltd., Wuhan) (solutions I, II, III, 10 min each), then dehydrated using graduated ethanol sequence (100% ethanol I, II, III, 5 min each). Sections were thoroughly rinsed with distilled water to remove residual ethanol. Thermal antigen retrieval was executed using sodium citrate buffer (pH 6.0) under constant temperature conditions, preventing buffer evaporation and tissue detachment. Thermal antigen retrieval was performed using EDTA buffer (pH 8.0) with a microwave-based procedure: medium power for 10 min, followed by a 5-min pause, then medium-low power for 5 min, another 2-min pause, and a final 5-min incubation at medium-low power. During retrieval, buffer evaporation and tissue desiccation were strictly prevented to avoid tissue damage. After retrieval, sections were naturally cooled to normal temperature. Sections underwent three washes with phosphate-buffered saline (PBS, pH 7.4) (5 min each), and reagent-confined areas were established around tissues using a hydrophobic barrier pen (Servicebio Biotechnology Co., Ltd., Wuhan). Blocking was carried out using 3% bovine serum albumin (BSA) under room temperature conditions for 30 min to decrease non-specific binding. Subsequently, the anti-Aβ42 primary antibody (rabbit-derived, Servicebio Biotechnology Co., Ltd., Wuhan; diluted 1:200) was added, and sections were incubated overnight in a humidity chamber at 4 °C. After three PBS cleanings (5 min each), fluorescently tagged secondary antibody was added and incubated for 50 min at room temperature under light-protected conditions. Cell nuclei were counterstained using DAPI (4’,6-diamidino-2-phenylindole) for 10 min. Sections underwent treatment with 0.3% Sudan Black B solution for 5 min to remove tissue autofluorescence. Following rinsing under tap water for 10 min, sections were mounted using anti-fade mounting medium (ProLong Gold, Thermo Fisher Scientific). Images were collected through an upright fluorescence microscope (Nikon Eclipse Ci, Nikon Corp.) and Aβ42 immunofluorescence intensity was quantitatively evaluated using ImageJ software (version 1.53k, National Institutes of Health, USA), including background subtraction and threshold-based region of interest analysis. The overall experimental workflow is illustrated in Fig. 1E.

### Data processing and statistical analysis

All local field potential (LFP) data were handled and analyzed through customized MATLAB 2021b scripts (MathWorks Inc., USA). Statistical analyses were completed using GraphPad Prism 9.0 software (GraphPad Software Inc.). Group differences in behavioral performance, power spectral density, phase-amplitude coupling, phase-locking values, and average Aβ42 immunofluorescence intensity were examined through one-way ANOVA or repeated measures ANOVA, then followed by Tukey’s method for multiple comparisons. Continuous variables are displayed as mean ± standard deviation (mean ± SD). Box plots depict the median, interquartile range (IQR), and whiskers extending to the most extreme values within 1.5×IQR. Statistical significance criteria were determined as: *P < 0.05, **P < 0.01, ***P < 0.001.

## Results

### Gamma-LFMS enhanced spatial memory performance in AD mice

**Fig. 2:**
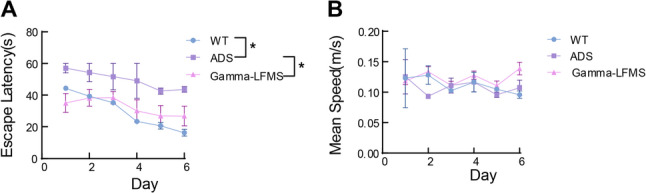
Effects of gamma-LFMS on spatial learning ability in AD mice. MWM test results: (**A**) Escape latency of WT, ADS, and gamma-LFMS groups over the 6-day training period. **B** Average swimming speed of WT, ADS, and gamma-LFMS group mice. Data are presented as mean ± SD; *indicates *p* < 0.05, *n* = 8/group

We first evaluated the effects of gamma-LFMS on spatial memory performance in AD mice using the Morris water maze test. Analysis of escape latency revealed that WT mice demonstrated significantly shorter latency compared to ADS mice, while gamma-LFMS led to a significant reduction in escape latency (*p* < 0.05) (Fig. 2). The performance (in terms of escape latency) of the three groups of mice in the Morris water maze task per day is shown in Table 1. No significant differences in average swimming speed were observed across groups (*p* > 0.05), excluding potential confounding effects of motor ability differences and confirming that observed latency changes reflected spatial memory capacity rather than motor function impairments.

**Table 1:** Mean ± Standard Deviation (SD) of Escape latency in Mice of Different Groups

Training Day	WT Group	ADS Group	gamma-LFMS Group
Day 1	44.34 ± 7.98	57.04 ± 3.01**	33.40 ± 16.42*
Day 2	39.30 ± 1.55	54.30 ± 8.05*	35.21 ± 13.87*
Day 3	35.25 ± 0.64	51.65 ± 11.77*	39.49 ± 10.60*
Day 4	23.40 ± 1.69	49.05 ± 15.31*	31.59 ± 18.93*
Day 5	20.70 ± 2.97	42.66 ± 2.79**	27.47 ± 19.24**
Day 6	16.30 ± 2.97	43.64 ± 2.55**	28.28 ± 16.85**

Notes next to the values of the ADS group: indicate the statistical significance of differences compared with the WT group; Notes next to the values of the gamma-LFMS group: indicate the statistical significance of differences compared with the ADS group

*indicates *p* < 0.05, **indicates *p* < 0.01

### Gamma-LFMS enhanced theta and gamma band power in AD mice

**Fig. 3:**
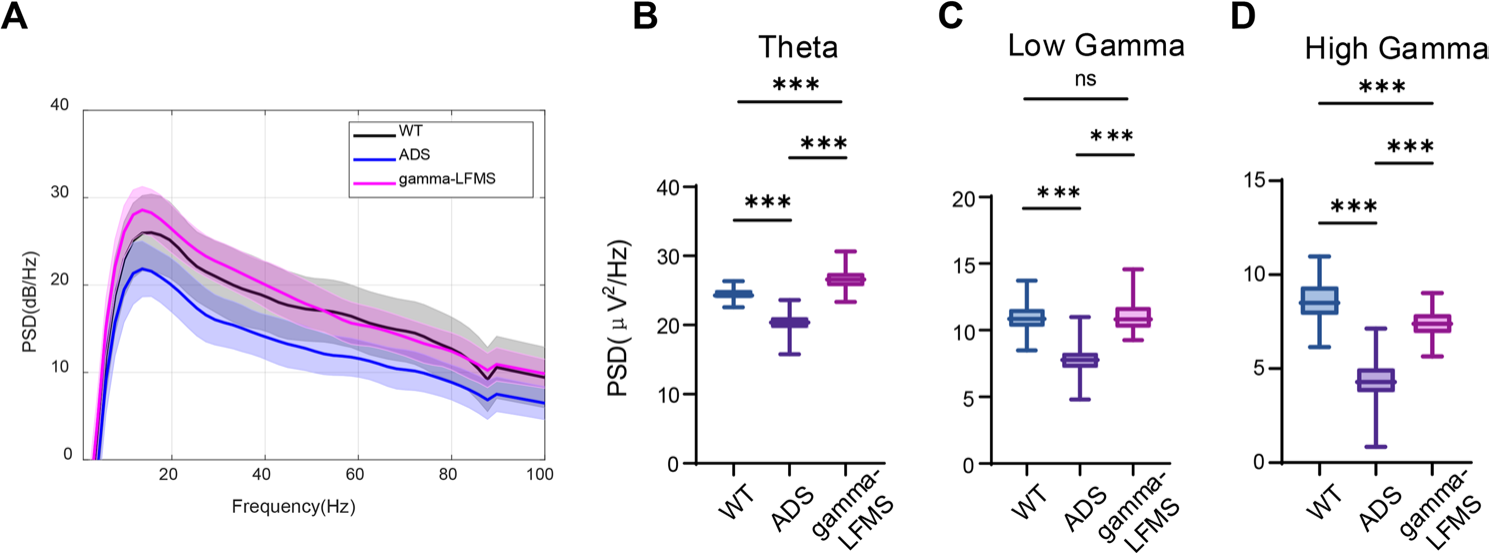
Effects of gamma-LFMS on theta and gamma band PSD in AD mice. **A** PSD curves of hippocampal CA1 region in WT, ADS, and gamma-LFMS group mice, with solid curves representing PSD mean values and light-colored areas representing variance of mean values. Statistical analysis plots of PSD for (**B**) theta (4–12 Hz), (**C**) low gamma (30–60 Hz), and (**D**) high gamma (60–100 Hz) bands. Box plots show the median, interquartile range (IQR), and whiskers. ***indicates *p* < 0.001; *n* = 8/group

We explored the regulatory impacts of gamma-LFMS on theta and gamma band power spectral density (PSD) in the hippocampal CA1 area of AD mice. PSD analysis demonstrated that both theta and gamma band PSD were reduced in ADS mice compared to WT controls (Fig. 3A), with statistically significant differences (*p* < 0.001) (Fig. 3B-D). Treatment with gamma-LFMS significantly enhanced PSD in both theta and gamma bands in AD mice (*p* < 0.001) (Fig. 3B-D). Following gamma-LFMS intervention, low gamma band PSD in AD mice approximated WT levels with no statistical significance (*p* > 0.05). Within the theta band, the gamma-LFMS group showed remarkably higher PSD than the WT group (*p* < 0.001), whereas within the high gamma band, the gamma-LFMS group exhibited remarkably lower PSD compared to WT controls (*p* < 0.001).

### Gamma-LFMS Improved Phase-Amplitude Coupling in Hippocampal CA1 Region of AD Mice

**Fig. 4:**
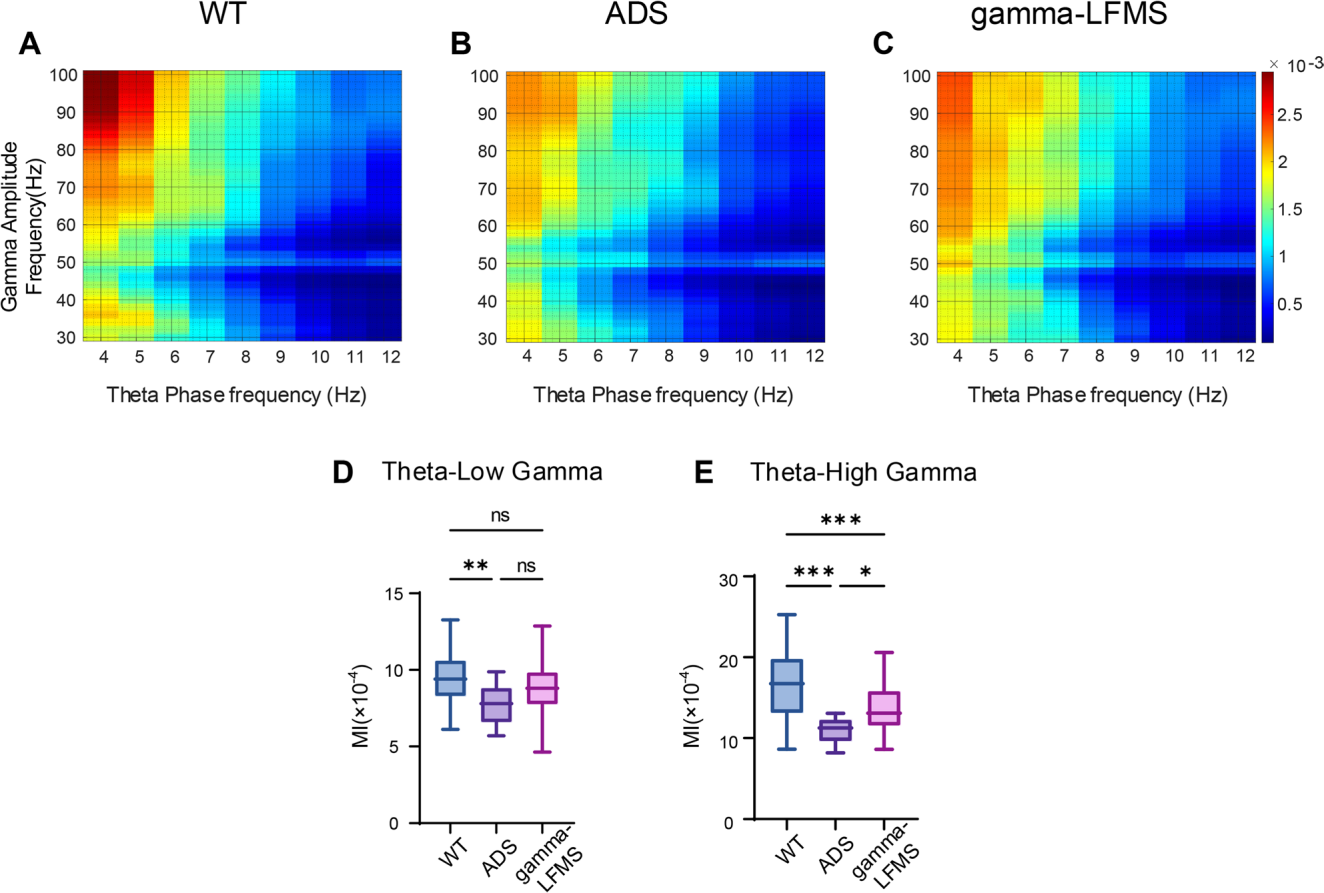
Effects of gamma-LFMS on PAC in hippocampal CA1 region of AD mice. Modulation index (MI) quantified phase-amplitude coupling in three groups of mice: (**A**), (**B**), and (**C**) are phase-amplitude coupling heatmaps, the horizontal axis represents the frequency of the theta phase, the vertical axis represents the frequency of the gamma amplitude, and the PAC strength is denoted by MI (Modulation Index). The closer the color is to red, the higher the MI value. **D** and **E** are statistical analysis plots of MI values for the three groups. Box plots show the median, interquartile range (IQR), and whiskers. *indicates *p* < 0.05, **indicates *p* < 0.01, ***indicates *p* < 0.001; *n* = 8/group

To investigate the effects of gamma-LFMS on neural rhythms in the CA1 region of the hippocampus in AD mice, we quantified phase–amplitude coupling (PAC) using the modulation index (MI; Fig. 4). Figures 4A–C present PAC for the three experimental groups, visualizing the coupling between theta-band phase and gamma-band amplitude. Across groups, gamma-band amplitude was preferentially coupled to phases in the 4–8 Hz range, and theta–high gamma coupling was stronger than theta–low gamma coupling. These heat maps provide a qualitative overview of PAC patterns, whereas the exact MI values are given by the statistical results in Fig. 4D and E. Statistical analysis showed that, relative to WT controls, AD mice exhibited significantly reduced theta–low gamma and theta–high gamma MI values (Fig. 4D, E). Gamma-LFMS partially restored PAC in AD mice, as evidenced by a significant increase in theta–high gamma MI values relative to untreated AD mice, although these values remained lower than those of WT controls (Fig. 4D, E). The therapeutic effect of gamma-LFMS was particularly pronounced for theta–high gamma coupling.

### Effects of gamma-LFMS on neural network synchrony in hippocampal CA1 Region

**Fig. 5:**
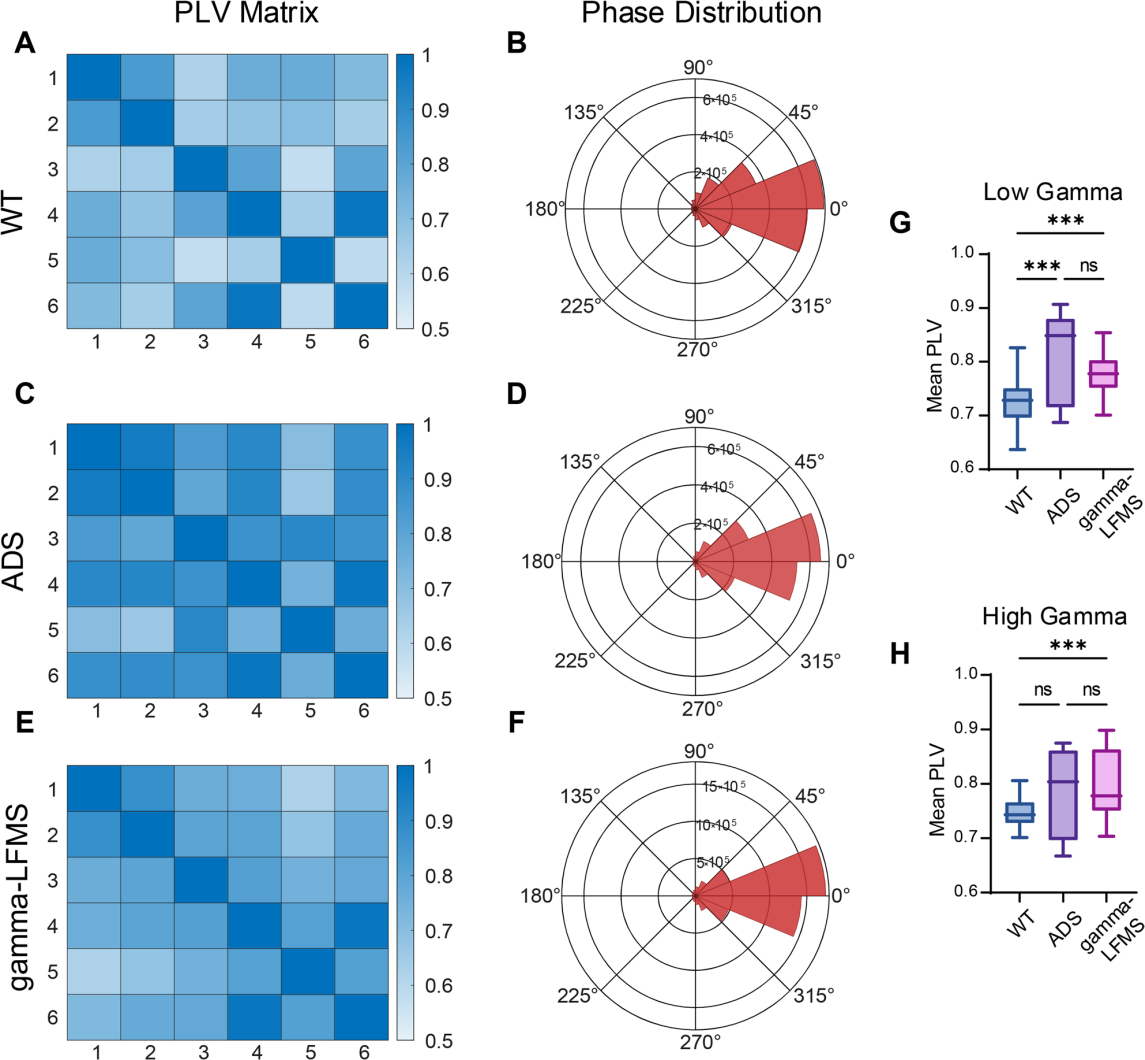
Effects of gamma-LFMS on neural network synchrony in hippocampal CA1 region of AD mice. Neural network synchrony quantified using phase locking value (PLV): (**A**) PLV Matrix and (**B**)phase distribution among 6 recording channels in the CA1 region of WT mice, within the gamma band (30–100 Hz), (**C**) and (**D**) for ADS group, (**E**) and (**F**) for gamma-LFMS group; (**G**) and (**H**) show the average PLV across all pairs of channels for the low and high gamma bands, respectively. Box plots show the median, interquartile range (IQR), and whiskers. ***indicates *p* < 0.001; *n* = 8/group

We employed phase locking value (PLV) analysis to investigate the modulatory effects of gamma-LFMS on hippocampal neural network synchronization properties within the 30–100 Hz frequency range. Results demonstrated that gamma-LFMS modulated neural synchrony in the hippocampal CA1 region of AD mice (Fig. 5). PLV matrix plots revealed that synchrony was elevated in ADS mice compared to WT controls (Fig. 5A, C, E). Phase distribution analysis showed that phases in all three groups were primarily distributed between 330° and 30° (Fig. 5B, D, F), with gamma-LFMS producing more concentrated phase distributions. Statistical analysis revealed a significant increase in the PLV in the low-gamma band in AD mice compared to the WT controls (*p* < 0.001) (Fig. 5G). Although gamma-LFMS treatment showed a trend by reducing the low-gamma PLV in AD mice, this change was not statistically significant (*p* = 0.09). Notably, the mean PLV in the gamma-LFMS group remained significantly higher than that in the WT group (*p* < 0.001). In the high-gamma band, the PLV was higher in AD mice than in WT controls (Fig. 5H), but this difference was not significant (*p* > 0.05). Gamma-LFMS did not significantly alter the high-gamma PLV compared to the untreated AD group (*p* > 0.05), and significantly higher compared to WT controls. These results suggest that while gamma-LFMS did not completely reverse the aberrant synchrony observed in AD mice, it induced a trend of desynchronization in the low-gamma range. The significant difference between the gamma-LFMS and WT groups may indicate that the stimulation modulates network activity towards, but not necessarily to, the baseline state.

### Gamma-LFMS reduced Aβ42 burden in the dentate gyrus of AD mice

**Fig. 6:**
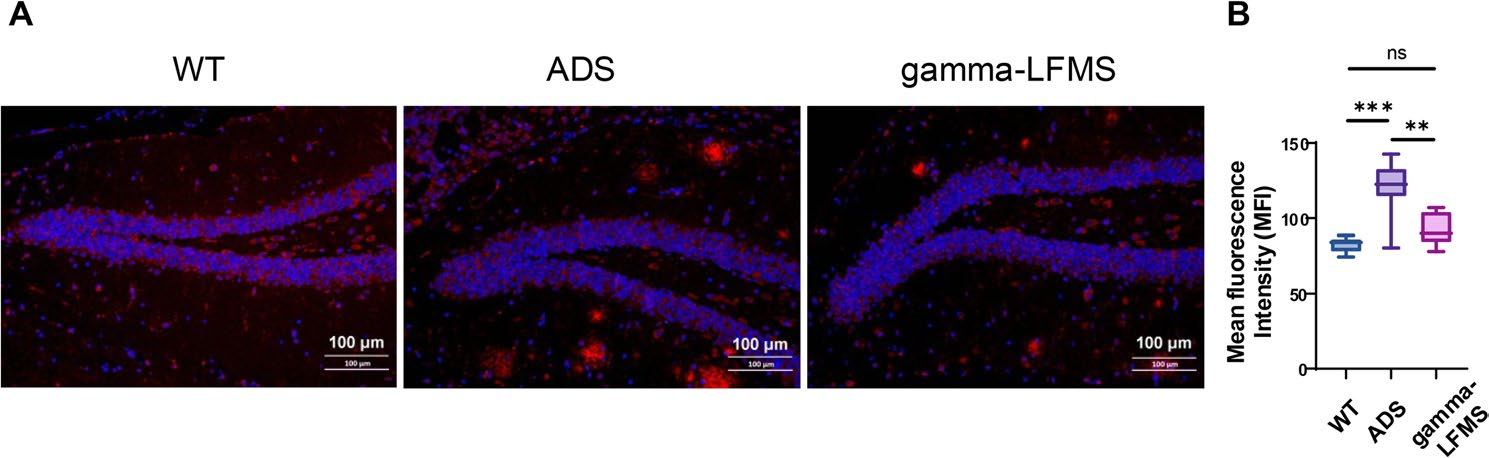
Representative immunofluorescence images showing Aβ42 (red) and DAPI (blue) in the dentate gyrus of WT, ADS, and gamma-LFMS group mice. **A** The entire DG region shown represents the ROI used for quantitative analysis, including the granule cell layer (characterized by densely packed DAPI-stained nuclei forming a characteristic C-shaped or wavy structure) and the molecular layer. Scale bar: 100 μm. **B** Statistical analysis of immunofluorescence results from three groups. Box plots show the median, interquartile range (IQR), and whiskers. **indicates *p* < 0.01, *** indicates *p* < 0.001; *n* = 8/group

Quantitative immunofluorescence analysis was performed to investigate the effects of gamma-LFMS on Aβ42 burden in the hippocampal dentate gyrus (DG) of APP/PS1 mice (Fig. 6). Figure 6A shows representative immunofluorescence images of Aβ42 immunoreactivity (red) and DAPI nuclear counterstaining (blue) in the dentate gyrus (DG). The region of interest (ROI) for Aβ42 quantification corresponds to the DG area shown in Fig. 6A, encompassing the granule cell layer and molecular layer. Aβ42 levels in the DG granule cell layer and molecular layer of sham-stimulated APP/PS1 mice were significantly elevated compared to wild-type controls (*p* < 0.001), reflecting substantial amyloid protein accumulation. Treatment with gamma-LFMS significantly reduced Aβ42 content (*p* < 0.01) (Fig. 6B).

## Discussion

In this study, we aimed to investigate the therapeutic effects of gamma-LFMS on spatial memory capacity, hippocampal CA1 neural oscillations, and pathological markers in AD mice. The key role of gamma-LFMS was demonstrated through significant improvement in spatial memory capacity of AD mice. Beyond the substantial reduction in hippocampal amyloid protein load, we also detected enhanced gamma band power spectral density and theta-gamma coupling in the hippocampal CA1 region, as well as decreased neural network hypersynchrony. Overall, this study provides the first evidence that gamma-LFMS reduces amyloid protein deposition and improves spatial memory capacity through modulation of gamma rhythm neural oscillations.

Following gamma-LFMS, AD mice showed significantly shortened escape latency when performing the MWM task (*P* < 0.05) (Fig. 2A), indicating marked improvement in spatial memory capacity. Importantly, the average swimming speed remained unchanged across all groups (*P* > 0.05) (Fig. 2B), confirming that this improvement reflected genuine cognitive recovery rather than motor interference. The hippocampal CA1 region is directly involved in spatial memory and serves as the spatial hub for mouse spatial memory capacity [26]. Disruption of CA1 neural activity can lead to impaired spatial memory capacity in mice [27, 28]. In APP/PS1 mice, alterations in neuronal activity and encoding within the CA1 region are primary factors underlying spatial memory deficits [29–31]. In this study, 18 days of gamma-LFMS resulted in restoration of low gamma power (30–60 Hz) to wild-type levels (Fig. 3C), indicating that gamma band power retains considerable plasticity even in established AD pathology. This finding is consistent with clinical observations that gamma activity, while diminished in AD patients, can be partially rescued through various interventions [32–34]. Furthermore, theta band power was also enhanced and exceeded WT levels (Fig. 3B), demonstrating that this intervention effectively reversed AD-related theta rhythm deficits. Conversely, despite gamma-LFMS treatment, high gamma band power (60–100 Hz) remained significantly lower than WT mice (Fig. 3D), indicating limited enhancement of high gamma band power by gamma-LFMS. Particularly noteworthy is that gamma-LFMS showed the best improvement effect on low gamma power, approaching WT levels. However, excessively elevated theta power may be detrimental, as neural networks function optimally in a balanced state rather than simply higher being better [35–37].

Compared with WT controls, ADS mice exhibited a marked reduction in both theta–low gamma and theta–high gamma PAC. This finding suggests a weakening of functional interactions between theta and gamma rhythms in CA1, reflecting reduced coordination among oscillations in different frequency bands. Gamma-LFMS partially restored PAC in AD mice: relative to untreated AD animals, gamma-LFMS–treated AD mice showed a significant increase in theta–high gamma modulation index (MI) values, although these values remained lower than those of WT controls (Fig. 4D, E). Previous studies have demonstrated that GABAergic interneurons are critical for the generation of theta and gamma oscillations and for their cross-frequency coupling, and that these cells are particularly vulnerable in AD [38]. Thus, we speculate that the attenuation of theta–gamma PAC in the CA1 region of AD mice may, at least in part, reflect dysfunction of local inhibitory interneuron networks. Whether gamma-LFMS improves cross-frequency coupling by modulating GABAergic interneurons, however, remains unclear and will require further investigation.

Our results showed that PLV in the low gamma band within hippocampal CA1 was significantly higher in AD mice than in WT mice, which is inconsistent with the findings of Sedghizadeh et al. [39] and Lahijanian et al. [40], who observed reduced neural synchrony in AD patients. This discrepancy may be attributed to our observation of network synchrony within hippocampal CA1, rather than phase synchrony between different brain regions. Gamma-LFMS reduced (trend) excessive neural synchrony within CA1, though without significant difference (Fig. 5G, H). In addition, previous work in AD model animals and patients has shown that abnormal network hyperexcitability and hypersynchrony in hippocampal circuits are associated with impaired information processing and cognitive dysfunction [41–45]. Taken together with these studies, our findings suggest that the hypersynchronous state observed in the CA1 region of AD mice may represent a maladaptive network configuration that constrains the flexibility of neuronal assemblies and, consequently, hinders normal information processing.

We hypothesize that this hypersynchronous state within hippocampal CA1 impairs information processing capacity and may contribute to cognitive decline. The gamma-LFMS-driven reduction in hippocampal Aβ42 (*P* < 0.01; Fig. 6) provides important translational evidence that 10 mT non-invasive gamma rhythm magnetic stimulation can ameliorate amyloid pathology. However, the underlying mechanisms remain unclear, with potential mechanisms including: (1) enhancing microglial phagocytosis of Aβ through microglial activation; (2) strengthening autophagy signaling pathways to promote Aβ degradation; (3) improving cerebrospinal fluid (CSF) and interstitial fluid (ISF) dynamics, enhancing glymphatic system clearance of metabolic waste products such as Aβ.

Our study has several limitations that warrant consideration. First, while our sample size is consistent with similar electrophysiological studies, future work would benefit from a larger cohort to enhance generalizability. Second, While the APP/PS1 transgenic model is well-established, it primarily recapitulates amyloid pathology without fully modeling the tau-related neurofibrillary tangles characteristic of human AD. Future studies should evaluate the efficacy of gamma-LFMS in models that better capture the full spectrum of AD pathophysiology, including 3xTg-AD and tau-focused models. Additionally, our analysis was primarily focused on hippocampal CA1, while AD involves distributed network dysfunction across multiple brain regions. Comprehensive mapping of stimulation effects across the broader limbic memory system would provide a more complete understanding of therapeutic mechanisms.

In conclusion, our study provides the first demonstration that gamma-LFMS can produce comprehensive intervention effects on cognition, neurophysiology, and pathology in AD mice. The restoration of specific frequency neural oscillations, optimization of cross-frequency coupling, modulation of pathological synchrony, and clearance of amyloid pathology collectively support the beneficial effects of gamma-LFMS in AD. Our findings provide experimental evidence and theoretical foundation for gamma-LFMS as a potential intervention for treating AD, and provide a theoretical foundation for developing cost-effective home-based prevention and treatment devices applicable throughout the lifespan.

## Supplementary Information

Supplementary Material 1: Figure S1. Atlas-guided localization of hippocampal CA1 and simulated distribution of the induced electric field magnitude (|E|, V/m) generated by gamma-LFMS. The |E| map was computed in COMSOL using the coil geometry and experimental positioning. CA1 is indicated based on the corresponding atlas level/segmentation. The map shows that induced electric fields extend to the depth of hippocampal CA1 under the stimulation parameters used in this study.

## Data Availability

The datasets generated and analyzed during the current study are available from the corresponding author upon reasonable request.
