# Supplementary figures and images for: Gamma low field magnetic stimulation ameliorates pathophysiological damage and cognitive impairments in AD mice
Source: Alzheimers Res Ther. 2026 Apr 18;18:131. doi: 10.1186/s13195-026-02052-1 (PMC13220417; doi:10.1186/s13195-026-02052-1)

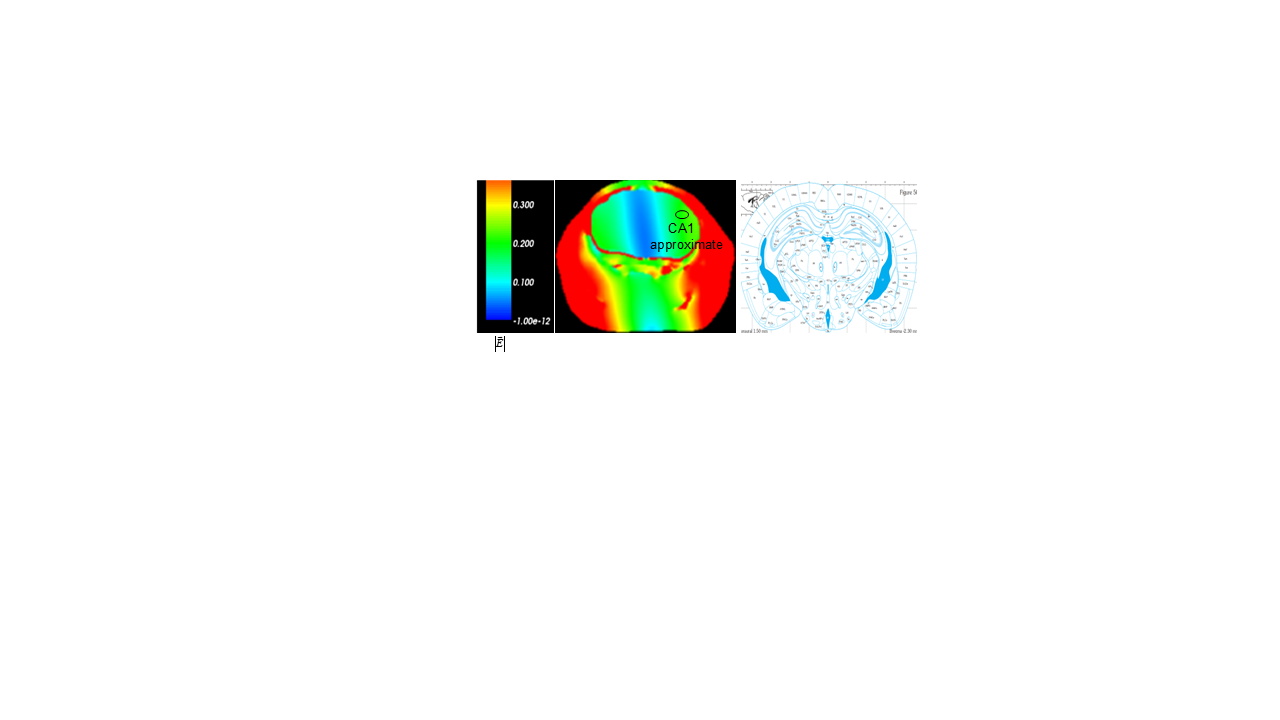

Supplement: Supplementary file 1 — Supplementary Material 1: Figure S1. Atlas-guided localization of hippocampal CA1 and simulated distribution of the induced electric field magnitude (|E|, V/m) generated by gamma-LFMS. The |E| map was computed in COMSOL using the coil geometry and experimental positioning. CA1 is indicated based on the corresponding atlas level/segmentation. The map shows that induced electric fields extend to the depth of hippocampal CA1 under the stimulation parameters used in this study. [file 13195_2026_2052_MOESM1_ESM.tif]
